# Retraction: Modeling and Experimental Analyses Reveals Signaling Plasticity in a Bi-Modular Assembly of CD40 Receptor Activated Kinases

**DOI:** 10.1371/journal.pone.0347622

**Published:** 2026-04-21

**Authors:** The PLOS One Editors

After this article [1] was published, concerns were raised about Figs 8, S6, and S7.

Specifically:

- In Fig 8B, lanes 2 and 3 of both the IM – GAPDH and IM – Lyn panels appear more similar than would be expected from independent results.
- The Fig S6A p-syk (1 µg/ml) and Fig S6D p38 (αCD40 -1 µg/ml) panels appear similar.
- The Fig S7B p38 and Fig S7F Syk panels appear similar.

The Corresponding Author did not agree with the above concerns and stated that the original image data underlying the figures of concern are no longer available. In the absence of the original image data, the concerns remain unresolved.

In light of the above unresolved concerns, which question the integrity and reliability of the reported results and conclusions, the *PLOS One* Editors retract this article.

BS did not respond to the final editorial decision. US, AS, MM, VK, RS, SP, NS, SR, SS, IG, AGC, and RM either did not respond directly or could not be reached.
